# Sea-Buckthorn Seed Oil Induces Proliferation of both Normal and Dysplastic Keratinocytes in Basal Conditions and under UVA Irradiation

**DOI:** 10.3390/jpm11040278

**Published:** 2021-04-07

**Authors:** Maria Dudau, Alexandra Catalina Vilceanu, Elena Codrici, Simona Mihai, Ionela Daniela Popescu, Lucian Albulescu, Isabela Tarcomnicu, Georgeta Moise, Laura Cristina Ceafalan, Mihail E. Hinescu, Ana-Maria Enciu, Cristiana Tanase

**Affiliations:** 1 Victor Babes National Institute of Pathology, Biochemistry, 050096 Bucharest, Romania; maria.dudau@ivb.ro (M.D.); elena.codrici@ivb.ro (E.C.); simona.mihai@ivb.ro (S.M.); daniela.popescu@ivb.ro (I.D.P.); lucian.albulescu@ivb.ro (L.A.); laura.ceafalan@ivb.ro (L.C.C.); mhinescu@yahoo.com (M.E.H.); cristianatp@yahoo.com (C.T.); 2 Faculty of Medicine, Carol Davila University of Medicine and Pharmacy, 050474 Bucharest, Romania; alexandra.vilceanu@gmail.com; 3 SC Cromatec Plus, 077167 Ilfov, Romania; isa.tarcomnicu@gmail.com (I.T.); georgeta.moise@scient.ro (G.M.); 4 Faculty of Medicine, Titu Maiorescu University, 031593 Bucharest, Romania

**Keywords:** sea-buckthorn seed oil, long-chain fatty acids, skin dysplastic keratinocytes, UVA, CD36, SR-B2

## Abstract

Past decades demonstrate an increasing interest in herbal remedies in the public eye, with as many as 80% of people worldwide using these remedies as healthcare products, including those for skin health. Sea buckthorn and its derived products (oil; alcoholic extracts), rich in flavonoids and essential fatty acids, are among these healthcare products. Specifically, sea buckthorn and its derivatives are reported to have antioxidant and antitumor activity in dysplastic skin cells. On the other hand, evidence suggests that the alteration of lipid metabolism is related to increased malignant behavior. Given the paradoxical involvement of lipids in health and disease, we investigated how sea-buckthorn seed oil, rich in long-chain fatty acids, modifies the proliferation of normal and dysplastic skin cells in basal conditions, as well as under ultraviolet A (UVA) radiation. Using real-time analysis of normal and dysplastic human keratinocytes, we showed that sea-buckthorn seed oil stimulated the proliferation of dysplastic cells, while it also impaired the ability of both normal and dysplastic cells to migrate over a denuded area. Furthermore, UVA exposure increased the expression of CD36/SR-B2, a long-chain fatty acid translocator that is related to the metastatic behavior of tumor cells.

## 1. Introduction

Past decades show an increasing interest in herbal remedies in the public eye, and almost 80% of the population worldwide is now using them as healthcare products [1,2], particularly in developing countries. Sea buckthorn (*Elaeagnus rhamnoides* L.) is a unique medicinal and aromatic plant, frequently used as part of various pharmaceutical treatments, some of which are related to skin care. Sea-buckthorn-derived alcoholic extracts and seed oil were tested for antioxidant, antitumor and regenerative properties [3,4]. Most of these properties are related to its complex structure, rich in flavonoids and essential fatty acids (FA) [5]. Antioxidant activity was suggested as a protectant against different types of irradiation (gamma irradiation [6], UVA and UVB [3]), and, indeed, sea-buckthorn oil treatment showed increased antioxidant protection in irradiated keratinocytes [3]. Anti-tumor activity of sea-buckthorn-derived products was mostly related to their phenolic compounds [7], mostly by inhibition of fatty acid synthase [8,9]. However, sea-buckthorn seeds and berries are rich in FA [10], which constitute an important percentage of the sea-buckthorn seed oil [5]. It is expected that various FA would contribute to the cellular effects reported in sea-buckthorn-oil-related studies, including effects on skin cells and in animal models.

Normal adult human skin contains a variable dysplastic cell population, which gradually increases with sun exposure [11,12], mostly following UV exposure. These cells are characterized by atypical nuclei and disorganized growth, leading to precancerous skin lesions [13]. Evidence demonstrates that UVA irradiation of dysplastic keratinocytes has detrimental effects in the first few hours of exposure, but long-term, it activates cell protection and survival mechanisms [14], aggravating their malignant potential. Additionally, malignancy was previously shown to be aggravated by alterations in cellular lipid metabolism [15,16] to favor cell survival and proliferation.

Given the paradoxical involvement of FA in health and disease, we investigated whether sea-buckthorn seed oil has regenerative properties for skin cells, in basal conditions as well as under UVA radiation, and if this effect is the same, regardless of the dysplastic nature of the cells. The expression of CD36/SR-B2, a fatty acid translocator, which favors the uptake of fatty acids from the extracellular environment, was also assessed in relationship to UVA exposure.

## 2. Material and Methods

### 2.1. Cell Lines and Cell Treatments

Normal human epidermal cells (HEKa-ATCC PCS 200-011) and dysplastic keratinocytes (DOK-ECACC) were cultivated in standard cell culture conditions (37°C, 5% CO_2_) according to manufacturer instructions regarding cell culture media and supplements. The sea-buckthorn oil was a cold-pressed, commercially available product of Romanian origin and stored as 1/10 DMSO solution. Further dilutions were made with complete cell culture medium and appropriate controls containing a comparable dilution of DMSO. Cells were exposed to UVA with a UV-365 nm lamp (VL-340 BLB model—Vilber Lourmat, France) at a light intensity of 381 µW/cm^2^, for 30 min, in a laminar flow hood, as previously described [17].

### 2.2. Sea-Buckthorn Seed Oil Fatty Acid Composition

Targeted quantification of fatty acids was carried out by liquid chromatography coupled with tandem mass spectrometry (LC-MS/MS). Analyses were performed on a triple quadrupole mass spectrometer model API3200 (Sciex) coupled with an Infinity 1260 binary pump (Agilent) and autosampler. Analyst software version 1.5.2 was used for data acquisition and processing. After testing several stationary phases, chromatographic separation was achieved on a Phenomenex Luna pentafluorophenyl (PFP2) column (100 mm × 2 mm, 3 μm, 100 Å) using a mobile phase composed of water and acetonitrile at a flow rate of 0.3 mL/min. Seed oil was dissolved in ethanol at 1 mg/mL, and 3 µL of the solution were injected in the LC-MS/MS system. The mass spectrometer was operated in negative electrospray ionization (ESI). Acquisitions were carried out in multiple reaction monitoring (MRM) mode using the specific traces of each fatty acid (see Supplementary information Table S1). A general screening of the seed oil volatile constituents was performed by gas chromatography–mass spectrometry (GC/MS). A Perkin Elmer CLARUS 680 gas chromatograph (equipped with an ELITE 5 MS column (1,4-bis (dimethylsiloxy)) phenylene dimethyl polysiloxane stationary phase, 30 m, 0.25 mm ID, 0.25 µm film thickness, eluted with helium at 1 mL/min), connected to a Clarus SQ8T quadrupole, was used for this purpose. For analysis, 1 µg of seed oil solutions prepared at 50 mg/mL in ethanol were injected in split mode (40:1). Compound identification was achieved by searching the obtained mass spectra in the NIST and Wiley libraries.

### 2.3. Cell Proliferation and Toxicity Assays

Cytotoxicity was assessed by cellular lactate dehydrogenase (LDH)release (CytoTox 96 Non-Radioactive Cytotoxicity Assay, Promega, Madison, WI, USA). Specifically, 10,000 cells were seeded in triplicate overnight in 96-well plates and incubated the next day with serial dilutions of stock solution for an additional 72 h. Two controls were included—a negative control (cell culture medium only) and a positive control (addition of Cell Lysis Reagent 10x, Promega, 45 min prior to LDH detection). For background subtraction, additional triplicates of cell-free wells were incubated with tested solutions. For LDH analysis, 50 µL of supernatant were collected from each well and incubated with assay reagent for 30 min in the dark. After the addition of stop solution, absorbance was read at 490 nm by using a microplate reader Anthos Zenyth 3100 (Wals, Austria). Cytotoxicity was assessed as a percentage of the positive control, according to the formula cytotoxicity = 100*(Sample OD − background OD)/(average positive control − average background).

Proliferation was assessed by MTS assay (CellTiter 96 AQueous One Solution Reagent, Promega, Madison, WI USA). Specifically, 10,000 cells were seeded in triplicates overnight in 96-well plates and incubated the next day with cell culture medium and/or serial dilutions of stock solution for 72 h. A positive control (non-treated cells) was included. For background subtraction, additional triplicates of cell-free wells were incubated with tested solutions. On the day of the reading, the cell media was removed, and 100 µL of fresh medium and 20 µL of MTS reagent were added to each well. The plate was incubated at 37 °C for 3 h in a humidified, 5% CO_2_ atmosphere. Absorbance was read at 490 nm by using a microplate reader Anthos Zenyth 3100 (Wals, Austria). Proliferation was assessed as a percentage of the control: % of proliferation = 100*(sample OD − background OD)/(average control − average control background).

Oil Red staining: Cells were fixed in 3.7% formaldehyde for 30 min to 1 h, washed with water, and then incubated with 60% isopropanol for 5 min. Oil Red O Stock solution (Sigma-Aldrich, St Louis, MO, USA) was reconstituted with 100% isopropanol. Oil Red O Working solution was prepared 15 min before staining by mixing three parts of Oil Red O Stock solution and two parts of water, and then the solution was filtered through Whatman No.1 filter paper. The cells were stained with Oil Red O Working solution for 10 min at room temperature and afterward washed 2–5 times with water until no excess stain was seen. DAPI staining can be performed after this step for 10 min, at room temperature, and protected from light. The red lipid droplets were visualized by using fluorescent microscopy with a Nikon TI (Nikon, Tokyo, Japan) inverted fluorescence microscope.

Real-time impedance readings: Cell adhesion and cell proliferation were assessed using RTCA DP platform (Agilent Technologies, Santa Clara, CA, USA). Cells were pre-irradiated with UVA for 30 min as previously described [17], and then they were trypsinized and seeded in E-16 plates at a density of 20,000 cells/well, with or without the cell medium, and supplemented or not with sea-buckthorn oil 1/8000. Readings were collected every 5 min for 24 h. Cell index at 2 h (at plateau) was used for statistical analysis (One-Way Anova, Dunnett multiple comparison, where data were compared to control. * *p* < 0.05, ** *p* < 0.01, *** *p* < 0.001, **** *p* < 0.0001)

Scratch-wound assay: Cells were seeded in a 35 mm glass-bottom dish and grown to confluence in normal cell culture conditions. A scratch was performed with a 200 µL pipette tip, with debris washed and cells further incubated in cell medium supplemented with 1/8000 sea-buckthorn oil for 24 h, in Nikon Biostation IM. Images were acquired with 20× Ph objective every 20 min, and data were analyzed with NIS-Elements BR (Nikon Tokyo, Japan).

### 2.4. Evaluation of the Expression CD36

Immunofluorescence (IF): Cells were grown on coverslips fixed with 4% formaldehyde for 5 min, rinsed in phosphate buffer saline (PBS), permeabilized in 0.1%Triton-X 100 and in 0.5% BSA for 10 min and incubated for 2 h with the primary antibody (anti-CD36 ThermoFisher PA1-16813, 1:100) at room temperature. After 3 × 5 min washes in PBS, cells were incubated with the secondary antibody (goat anti-rabbit Oregon Green 488, Invitrogen 011038, 1:2000 in PBS), and the nuclei were counterstained with DAPI. Image acquisition was performed with a Leica TCS SP8 white light laser confocal microscope.

Western blotting (WB): Cells were lysed in RIPA buffer with 1% protease inhibitor cocktail (P8340 Sigma), centrifuged for 10 min at 11,000 rpm (Ohaus R5510), and the supernatant boiled for 10 min (1:1 *v*/*v*) in Laemmli two times. Then, 40 µg of protein were loaded on each well, migrated in 10% SDS-PAGE gel for 2 h at 20 mA/gel and transferred onto a polyvinylidene fluoride (PVDF) membrane (BioRad) (1 h, 100 V). The membrane was incubated overnight in primary antibodies (anti-CD36 AF2519, R&D Systems, anti-GAPDH PA1-987, ThermoFisher), washed three times in TBS-T 0.5% and one time in TBS the next day and incubated for 1 h at room temperature (RT) in secondary antibodies (chicken-anti goat sc2953, SantaCruz, goat-anti rabbit 92680011, LiCor). Image acquisition was performed using a c-Digit scanner (LI-COR).

## 3. Results

### 3.1. Biochemical Composition of Sea-Buckthorn Seed Oil

**Figure 1:**
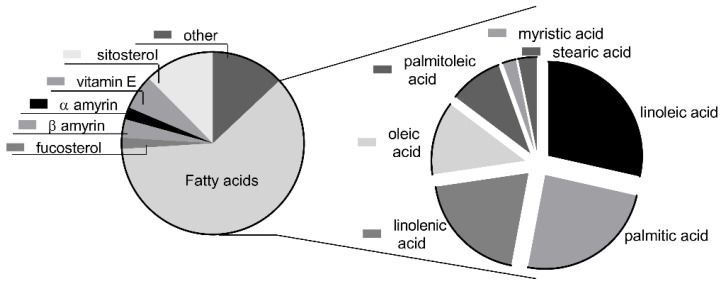
The chemical composition of the volatile fraction of sea-buckthorn seed oil, as assessed by gas chromatography–mass spectrometry.

Considering the important role of polyunsaturated fatty acids in skin growth and protection, we conducted a few experiments in order to estimate the composition of the tested sea-buckthorn seed oil. Linoleic acid was the main component (28.5% of the total fatty acid content), followed by palmitic acid (24.4%), linolenic acid (19.7%), oleic acid (12.6%) and palmitoleic acid (9.2%). Only small amounts of myristic and stearic acid were found in the tested oil (2.3 and 3.2%, respectively). A general screening of the volatile constituents of seed oil was also performed by using gas chromatography–mass spectrometry (GC/MS). Based on the total peak area, fatty acids represented 61% of the volatile compounds, with the other main components being sitosterol (12.4%), vitamin E (6.2%), α- and β-amyrin (2.14 and 3.3%, respectively) and fucosterol (1.96%) (Figure 1). The distribution of fatty acids was similar to that assessed by LC/MS.

### 3.2. The Sea-Buckthorn Seed Oil Showed Pro-Proliferative Effects at Low Concentrations

**Figure 2:**
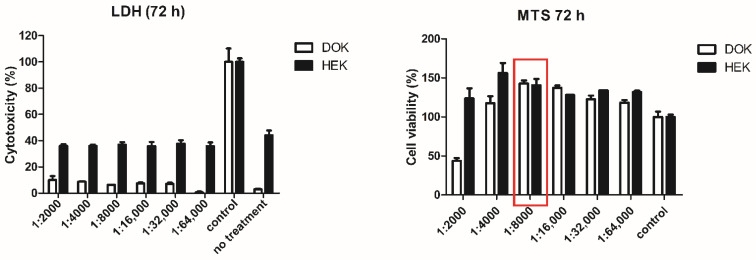
Assessment of toxicity of sea-buckthorn seed oil. Normal human keratinocytes (HEK) and dysplastic keratinocytes were seeded at a density of 10,000 cells/well in 96 well plates and treated for three days with the indicated dilutions of sea-buckthorn seed oil. Cytotoxicity was assessed by lactate dehydrogenate (LDH) release in the cell medium and viability by MTS assay. LDH control is represented by the lysis control (100% mortality). The MTS control was represented by cells maintained in standard cell culture conditions. The highest non-toxic and pro-proliferative concentrations (highlighted in red) were chosen for further tests. Bars represent average of triplicates, calculated as percentage to control.

We tested the effect of sea-buckthorn seed oil on viability of normal and dysplastic skin cells using serial dilutions starting at 1/2000 (*v*/*v* in complete cell medium). The starting concentration was chosen following previous experiments, in which higher concentrations were cytotoxic for both cell types (data not shown). Further dilutions showed no cytotoxicity when reported on non-treated controls. Cell viability increased with subsequent dilution up to 1/8000, and then it started to decrease for both cell types (Figure 2). As a result, this concentration was selected for further tests.

### 3.3. Sea-Buckthorn Seed Oil Inhibits Cell Migration of Dysplastic Keratinocytes

**Figure 3:**
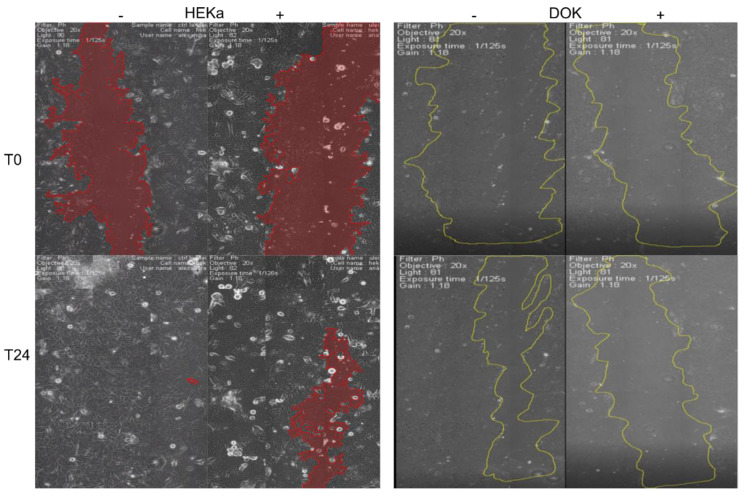
Real-time assessment of cell migration and proliferation of normal and dysplastic keratinocytes, treated (+) with sea-buckthorn seed oil. Treated cells (both normal and dysplastic) were less efficient in covering the denudated area. The effect was more prominent for dysplastic cells.

Next, in real-time microscopy we assessed the ability of cells to proliferate and migrate in a scratch-wound assay, following 24 h treatment with sea-buckthorn oil at 1/8000 dilution. After 24 h, both treated and untreated dysplastic cells failed to migrate and close the denuded area, and the oil treatment impaired migration even further (Figure 3). Normal keratinocytes were also less effective at closing the gap in the presence of diluted sea-buckthorn oil, but the effect was less impaired than that of dysplastic cells.

To this point, we noticed that there is a contrast between the effects of sea-buckthorn seed oil on cell proliferation versus cell migration, which might indicate a possible deleterious effect of such treatment on the migration of epidermis cell populations. We wanted to further confirm these findings and address other literature-reported effects of sea-buckthorn oil, such as protection against UVA irradiation and use of a real-time assessment of cell proliferation.

### 3.4. Treatment with Sea-Buckthorn Oil Following UVA Irradiation Does Not Mitigate the Deleterious Effect on Dysplastic Cells

**Figure 4:**
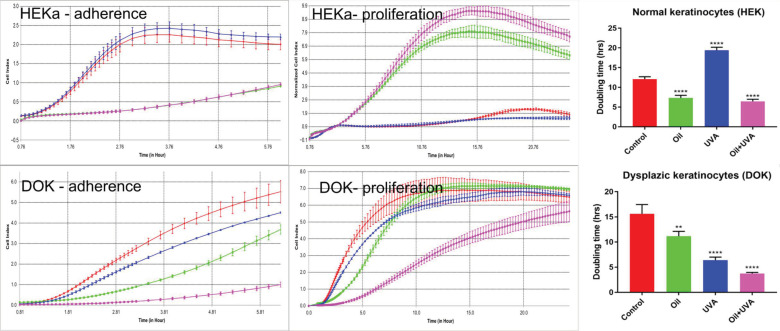
Cell adhesion and proliferation of normal and dysplastic keratinocytes, treated with sea-buckthorn seed oil, in the presence or absence of UVA irradiation. Each point on graphs represents the average cell index of triplicates, recorded every 15 min for 24 h. Bars represent average of triplicates with SD. One-Way Anova was used to assess statistical significance (** *p* < 0.01, **** *p* < 0.0001).

As sea-buckthorn oil is reported to have protective effects against irradiation, we tested the effect of UVA irradiation on normal and dysplastic keratinocytes adherence using a real-time impedance reading system. This investigation allowed for the quantification of cell adherence (e.g., required for re-adhesion of daughter-cells after cell division) and of cell proliferation by calculation of doubling times using the xCELLigence platform (Figure 4). Consistent with videomicroscopy observations, oil treatment impairs cell adhesion and fails to mitigate UVA irradiation effects. Doubling times analysis confirmed MTS data regarding stimulation of cell viability with the selected dilution of sea-buckthorn seed oil. Notably, oil treatment on irradiated cells significantly decreased doubling times, which translates into increased proliferation rate of cells.

### 3.5. UVA Irradiation Does Not Affect Uptake of Lipids into Normal and Dysplastic Keratinocytes

**Figure 5:**
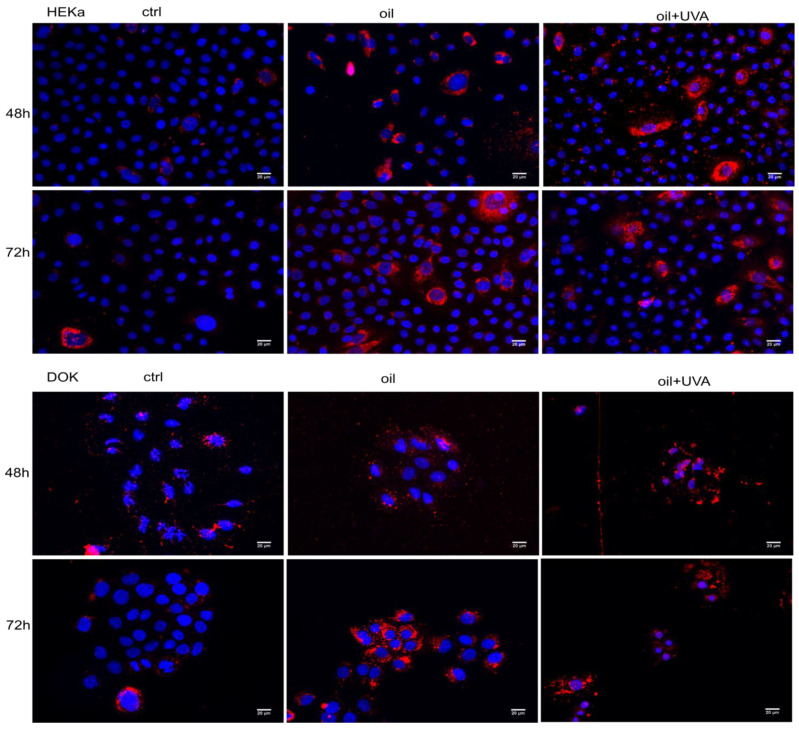
Oil uptake in irradiated and non-irradiated cells. Irradiated and non-irradiated cells were treated with sea-buckthorn seed oil (dilution 1/8000) for the indicated periods of time. Non-treated, non-irradiated cells were used as controls. Lipid inclusions were stained with Oil Red O.

Next, we tested whether irradiation affects oil uptake into cells. Keratinocytes use lipids to seal the epidermis from the outer environment by deposition of sphingolipids in the extracellular space [18]; therefore, a normal degree of lipid inclusion is to be expected in non-treated cells. Both normal and dysplastic cells uptake presented increased lipid load following oil treatment. However, unlike normal cells, where only some cells are loaded with lipid inclusions, all dysplastic cells have a degree of lipid load. Irradiation did not significantly affect the upload of lipids in both normal and dysplastic cells, but dysplastic cells showed a more uniform lipid uptake (Figure 5).

### 3.6. The Fatty Acid Translocator CD36 Is Expressed in Dysplastic, but Not Normal Keratinocytes

**Figure 6:**
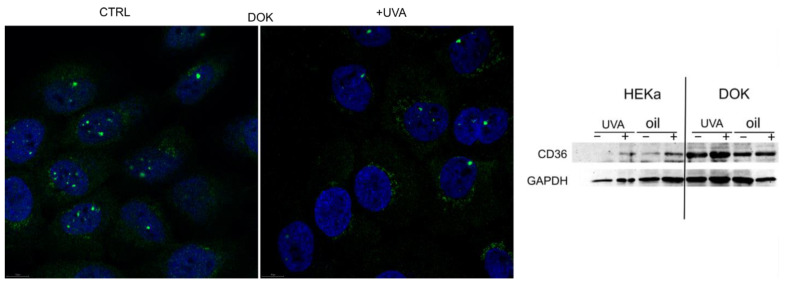
CD36 expression in normal (HEKa) and dysplastic (DOK) keratinocytes after UVA irradiation. Expression of CD36 in both HEKa and DOK cells was assessed by confocal immunofluorescence and confirmed by Western blot. In basal conditions, CD36 expression in normal keratinocytes is undetectable with the selected methods. Its expression increases in dysplastic cells, as well as following UVA irradiation.

Long-chain FA used by cells to synthesize phospholipids and sphingolipids do not easily diffuse through cell membranes and are more efficiently up-taken with the help of fatty acid translocators, such as CD36/SR-B2. Furthermore, CD36/SR-B2 was shown to be involved in the tumorigenesis of aggressive, metastatic cancers (reviewed in [19]). Using two different methods, we investigated the expression of CD36 in normal and dysplastic keratinocytes following UVA irradiation. The expression of CD36 in normal keratinocytes is undetectable in both IF (data not shown) and WB, and it appears to be slightly induced by irradiation. In contrast, dysplastic keratinocytes express a stronger signal for CD36, in both control and irradiated cells (Figure 6).

## 4. Discussion

Our manuscript compares the effect of sea-buckthorn seed oil on normal and dysplastic human keratinocytes, starting from the premise that normal skin contains a mixture of both cells, whose proportion changes with age. It was demonstrated that sea-buckthorn fatty acids have the ability to improve the post-inflammatory response resulting from deleterious UV exposure. Moreover, they could alleviate the effects of sun burns, support regenerative processes of the skin and appease irritation [5]. UVA exposure was reported to affect these cell populations differently in terms of migration and proliferation. It was previously demonstrated that dysplastic cells are less able to spread across a denuded area than normal keratinocytes, and were more affected than the latter by UVA treatment [17]. In terms of cell proliferation, UVA exposure induced cell-cycle arrest for normal keratinocytes [20,21]; this outcome, reviewed in [22], may lead to an increased doubling time of a cell population, a finding which also was observed in our real-time, impedance-reading experiments. Dysplastic cells do not exhibit this protective effect; they are more prone to proliferate under UV irradiation, and the seed-oil treatment further aggravates this noxious behavior.

One putative mechanism for increased dysplastic cell proliferation following seed -il treatment could be CD36-mediated lipid uptake. Fatty acid transporters, including CD36, are overexpressed in tissues with an increased fatty acid metabolism [23]. FA are able to activate peroxisome proliferator-activated receptors (PPARs) [24], transcription factors which further activate gene transcription of CD36 [25]. It was previously shown that tumor cells modify their lipid metabolism [15,16] to favor cell survival and proliferation. A study by Pascual et al. revealed that CD36+ cells react to dietary lipids and rely on lipid metabolism for metastatic potential. In their study, CD44 bright cells isolated from human oral carcinomas exerted a distinctive ability to overexpress both the fatty acid receptor CD36 and lipid metabolism genes, thus accelerating the initiation of metastasis [26].

Various studies in the literature shed light on the potential relationship between an impaired lipid metabolism and different skin conditions, but the extent to which fatty acid transporters are involved in directing fatty acids to keratinocytes, altering the fate of lipid metabolism, remains an open question [23]. As far as the current literature goes, normal keratinocytes do not require CD36 for their metabolism; it is only transiently expressed in pathological circumstances such as wounds [27], infectious [28] and autoimmune [29,30] cutaneous diseases and tumor-related pathologies [31]. A stable CD36 expression can be a driver for increased lipid metabolism, which can fuel a possible malignant transformation.

## 5. Conclusions

Plant-derived bioactive compounds or mixtures have gradually become a hot topic in recent years, as a growing body of data points to their beneficial effects. However, these effects can vary from cell type to cell type. Here, we showed that, although sea-buckthorn seed oil has been associated with skin health and protection against irradiation, it also stimulates proliferation of dysplastic cells, while impairing the ability of both normal and dysplastic cells for wound-healing. We also investigated the expression of CD36, a fatty acid translocator, on normal and dysplastic keratinocytes, and we found that UVA exposure increases its expression, which could hold functional significance for further progression towards malignancy.

## Supplementary Materials

The following are available online at https://www.mdpi.com/article/10.3390/jpm11040278/s1, Table S1: Optimized parameters for fatty acids mass spectrometric detection.
