# Supplementary material for: Sea-Buckthorn Seed Oil Induces Proliferation of both Normal and Dysplastic Keratinocytes in Basal Conditions and under UVA Irradiation
Source: J Pers Med. 2021 Apr 7;11(4):278. doi: 10.3390/jpm11040278 (PMC8067731; doi:10.3390/jpm11040278)
Supplement: Supplementary file 1 [file jpm-11-00278-s001.pdf]

Supplementary information

Table 1. Optimized parameters for fatty acids mass spectrometric detection

Compound	Q1 (Da)	Q3 (Da)	Scan time (msec)	CE (collision energy)	CXP (collision extraction potential)
Linolenic acid	277.218	277.218	40	−10	−6
	277.218	59.0	40	−30	−6
Linoleic acid	279.241	279.241	40	−14	0
	279.241	261.3	40	−16	0
Myristic acid	227.194	227.194	40	−36	−8
	227.194	78.8	40	−36	−8
Oleic acid	281.282	281.282	40	−10	0
	281.282	263	40	−22	0
Palmitic acid	255.251	255.251	40	−10	0
	255.251	237.0	40	−30	0
Palmitoleic acid	253.201	253.201	40	−10	−2
	253.201	235.0	40	−20	−2
Stearic acid	283.348	283.348	40	−10	−2
	283.348	265.2	40	−18	−2
